# Difficult to Treat Focal, Stiff Person Syndrome of the Left Upper Extremity

**DOI:** 10.1155/2017/2580620

**Published:** 2017-10-25

**Authors:** Nathan E. Esplin, John W. Stelzer, Timothy B. Legare, Sayed K. Ali

**Affiliations:** ^1^University of Central Florida College of Medicine, 6850 Lake Nona Boulevard, Orlando, FL 32827, USA; ^2^Department of Internal Medicine, Orlando VA Medical Center, 13800 Veterans Way, Orlando, FL 32827, USA

## Abstract

**Background:**

Stiff person syndrome (SPS) is a rare neurologic disorder characterized by muscle rigidity. It is a disorder of reduced GABA activity leading to increased muscle tone and often painful spasms. It generally presents in the axial musculature but rarely can involve only one limb, typically a lower extremity. In rare cases it can be paraneoplastic which generally resolves on treatment of the underlying neoplasm.

**Case Report:**

A 46-year-old male with a history of Hodgkin's Lymphoma in remission presented with left upper extremity pain secondary to a diagnosis of Stiff Person Syndrome limited to his left upper extremity. He had previously benefitted from plasmapheresis and was on diazepam and baclofen at home with relatively good control of his symptoms. SPS had previously been diagnosed with EMG and anti-GAD-65 antibody titers and was confirmed by an elevated anti-GAD-65 antibody titer. He was treated with plasmapheresis and maximum doses of medical treatment including botulinum toxin with only transient mild improvement in his symptoms.

**Conclusion:**

This case represents a case of a rare disease that was refractory to all known therapies. It outlines the need for further understanding of this disorder in order to provide better symptomatic treatment or potentially more definitive care.

## 1. Introduction

Stiff person syndrome (SPS) was first described in an “index” patient and 13 others in 1956 by Moersch and Woltman [1, 2]. SPS is a rare syndrome with an estimated incidence of one in one million, without racial preference, which follows the traditional pattern seen in autoimmune disease, affecting females in a 2 : 1 ratio to males [3, 4]. The clinical manifestations of SPS include features of both axial and appendicular muscular rigidity caused by involuntary motor unit activation and sensitivity [5]. The resultant rigidity most commonly evolves from the thoracolumbar paraspinal muscles and gradually extends to include proximal leg, and abdominal wall musculature, which leaves the patient with a stiff, hyperlordotic gait thereby giving the disease its namesake [6]. Additionally, patients are afflicted with episodic muscle spasms that are often preceded by external stimuli such as touch and loud sounds [6].

The primary pathophysiology in SPS is thought to be antibodies to the glutamic acid decarboxylase (GAD) inhibiting the rate-limiting step of *γ*-aminobutyric acid (GABA) [4, 7]. Decreased inhibitory GABA is considered to be the cause of the stiffness and spasms. Treatment centers on the two primary proposed pathophysiological mechanisms: a decreased GABA inhibitory response and autoimmunity. The first-line therapy has been to use GABA-A agonists, benzodiazepines, for the symptomatic relief of rigidity and spasms [6, 8]. Additionally, GABA-B agonists such as baclofen have been used to reduce muscle spasms, given either orally or intrathecally. Baclofen has relatively low CSF penetration given orally, but intrathecal administration is cautioned against because of the high risk of catheter infection and death due to autonomic instability [9]. Intravenous immunoglobulin (IVIG) and steroids have been relatively effective in reducing the symptoms as well, with better response in patients with anti-GAD specific SPS [9]. In a few cases in the literature botulinum toxin has been used in SPS or the focal variant, Stiff Limb Syndrome (SLS). It appears to have a relatively good effect with an improvement in symptomatology reported in the first few days after treatment, but it needs to be redosed every few months [6, 10, 11]. Finally, a new emerging treatment is the use of CD-20 inhibitor, rituximab. A randomized, controlled trial recently showed that rituximab benefitted some SPS patients, but only 26 patients were recruited for the study [12].

Here we present a case of anti-GAD-65 positive, treatment resistant, focal, stiff person syndrome confined to the left upper extremity in a patient with a medical history of Hodgkin's Lymphoma.

## 2. Case Presentation

Our patient is a 46-year-old male with a history of Hodgkin's Lymphoma diagnosed approximately 8 years before admission in remission status after rituximab therapy. He presented with subsequent left upper extremity focal SLS diagnosed two years before this admission by anti-GAD antibody elevation and electromyography (EMG) findings. He was recently on a maintenance regimen of rituximab therapy every 8 weeks but this had been discontinued approximately three months prior to this admission. He presented with left upper extremity pain, stiffness, and firm muscles. He was being managed by neurology with diazepam, hydrocodone, and tizanidine and reported two prior admissions in the last year for exacerbations of his SPS, once complicated by left upper extremity deep venous thrombosis. On both admissions, he was treated with plasmapheresis with little to no appreciable improvement of his symptoms but with significant reductions in anti-GAD-65 antibody titers.

On admission, the patient was found to have a limited range of motion in his left upper extremity with rigidity and apparent contracture with swelling secondary to a deep venous thrombosis. He was able to flex and extend his second and third digit, but was unable to move the fourth and fifth. Sensation was intact in all extremities, and his physical exam was otherwise unremarkable.

Lab workup was notable for an anti-GAD-65 antibody level of 64 IU/mL (reference range <5 IU/mL) by ELISA, a TSH of 0.240 mIU/L (reference range 0.35–4.94 mIU/L), and a C-PEPTIDE of 5.19 ng/mL (reference range 0.80–3.85 ng/mL). Previous EMG studies had demonstrated continuous motor unit activation of the left upper extremity consistent with a diagnosis of SLS, but the patient refused EMG and nerve conduction studies on this admission. He also refused further lab work to include anti-amphiphysin testing and lumbar puncture for CSF analysis.

A “mediport” catheter was placed and plasmapheresis was initiated on hospital day two with repeat plasmapheresis every other day for five total rounds. On hospital day one, he was started at 20 mg of diazepam four times per day, 12 mg of tizanidine every eight hours, and acetaminophen/hydrocodone three times per day as needed. By hospital day five he had had his second round of plasmapheresis, and his medicine regimen had been increased to 30 mg of diazepam every six hours, 10 mg of baclofen twice per day, 500 mg of divalproex twice per day, 9 mg of tizanidine every six hours, topical diclofenac gel twice per day, and 500 mg of methocarbamol every eight hours, still with poor control of his symptoms.

Neurology recommended the addition of botulinum toxin therapy, and on hospital day seven he was treated with 300 units of botulinum toxin type A (Xeomin), with 130 units injected in the left biceps, 70 units in the left brachioradialis, and 50 units injected into both flexor digitorum superficialis and profundus. On the following day, the patient reported some mild relief of his pain, but no relief of his rigidity, and on hospital day nine the patient stated both the pain and rigidity had both returned completely. Medical cannabis was considered, but was not an option per hospital policy.

This patient was discharged on hospital day ten with no further relief of his symptoms on maximal medical therapy and after his fifth round of plasmapheresis. He was discharged on 9 mg of tizanidine four times per day and 30 mg of diazepam four times per day. He was given instructions to follow up with neurology outpatient for assessment of his symptoms after botulinum injection and for repeat injections in three months. Approximately 14 days and again at 30 days after discharge contact was made with this patient by phone, and he advised on both occasions that he had no improvement in his symptoms. He declined further botulinum injection therapy, but opted to reestablish rituximab therapy with his oncologist. He refused follow-up EMG, NCS, and anti-GAD laboratory studies.

## 3. Discussion

SPS is a rare autoimmune, paraneoplastic, or cryptogenic disorder characterized by stiffness of either proximal trunk muscles or a specific limb or set of limbs and often by the presence of anti-GAD antibodies. GAD is the rate-limiting enzyme in the synthesis of GABA. There are two isoforms of GAD: GAD-65 and GAD-67. GAD-65 is found in the central nervous system, while GAD-67 is also found in other body systems. GAD-67 appears to be responsible for creating a basal level of GABA, while GAD-65 appears to be responsible for creating higher levels of GABA in response to stressors [2]. Anti-GAD-65 antibodies are found in around 80% of SPS, while anti-GAD-67 antibodies are found in around 60%. It is not clear if these antibodies are directly responsible for the disease, and other antibodies are associated with SPS and its variants. It is worth noting that the level of anti-GAD antibody does not correlate with clinical symptoms and does not reliably decrease in response to treatment, making it unreliable to assess effectiveness of therapy [2, 13].

The classic form of SPS involves increasing stiffness of the trunk and proximal muscles with painful muscle spasms. There are no other neurological symptoms other than the increased muscle tone and spasms, and the spasms can be severe enough to fracture bone [9]. It is an autoimmune disorder often associated with diabetes mellitus (DM) type I, and a variety of other autoimmune disorders. Like many autoimmune disorders, it is more common in women than men [13]. The focal, formerly called partial, form, Stiff Limb Syndrome (SLS) is also sometimes associated with anti-GAD antibodies, and usually involves one lower extremity [14]. This variant may eventually spread to the trunk. The etiology in most SPS (and SLS) cases is an autoimmune process, but in rare cases (approximately 5%) it is paraneoplastic, and fewer cases still are cryptogenic. Paraneoplastic SPS and SLS can also be associated with anti-GAD antibodies, but a majority of paraneoplastic cases are anti-GAD antibody negative [2, 9, 14, 15].

The prognosis of SPS and SLS is guarded, but most patients respond relatively well to treatment. All forms are generally responsive to benzodiazepines, while some cases require the addition of baclofen, IVIG, or plasmapheresis. There have been a few cases in the literature that required the use of methocarbamol, valproate, or eventually botulinum toxin to achieve good symptom control and even remission [10].

## 4. Conclusion

Our case demonstrates a case of anti-GAD-65 antibody positive, focal SLS of an upper extremity that has been resistant to benzodiazepines, baclofen, tizanidine, divalproex, rituximab, methocarbamol, multiple rounds of plasmapheresis, and botulinum toxin injection.
